# Applications of High-Throughput Sequencing for In Vitro Selection and Characterization of Aptamers

**DOI:** 10.3390/ph9040076

**Published:** 2016-12-10

**Authors:** Nam Nguyen Quang, Gérald Perret, Frédéric Ducongé

**Affiliations:** 1 CEA, DSV, I²BM, Molecular Imaging Research Center (MIRCen), 18 route du panorama, 92260 Fontenay-aux-Roses, France; nam.nguyenquang@cea.fr; 2 Neurodegenerative Diseases Laboratory, Centre National de la Recherche Scientifique (CNRS), Université Paris-Saclay, Université Paris-Sud, UMR 9199, 92260 Fontenay-aux-Roses, France; 3 LFB Biotechnologies, 3 avenue des Tropiques, 91958 Courtaboeuf CEDEX, France; perretg@lfb.fr

**Keywords:** high-throughput sequencing, SELEX, aptamers, evolution, fitness landscape

## Abstract

Aptamers are identified through an iterative process of evolutionary selection starting from a random pool containing billions of sequences. Simultaneously to the amplification of high-affinity candidates, the diversity in the pool is exponentially reduced after several rounds of in vitro selection. Until now, cloning and Sanger sequencing of about 100 sequences was usually used to identify the enriched candidates. However, High-Throughput Sequencing (HTS) is now extensively used to replace such low throughput sequencing approaches. Providing a deeper analysis of the library, HTS is expected to accelerate the identification of aptamers as well as to identify aptamers with higher affinity. It is also expected that it can provide important information on the binding site of the aptamers. Nevertheless, HTS requires handling a large amount of data that is only possible through the development of new in silico methods. Here, this review presents these different strategies that have been recently developed to improve the identification and characterization of aptamers using HTS.

## 1. Introduction

Nucleic acids can not only store genetic information but also form a multitude of three-dimensional structures to promote catalytic activity or interaction with different partners such as proteins. Since 1990, clever combinatorial approaches were developed to use these properties. The nucleic acid-based ligands found by these techniques were baptized by Ellington and Szostak [1]: aptamers, from the latin ‘aptus’ meaning ‘to fit’, and the method was popularized by the term Systematic Evolution of Ligands by Exponential enrichment (SELEX) by Tuerk and Gold [2]. The SELEX procedure has been extensively reviewed and consists in a succession of steps [3,4,5]. (1) A library of sequences is produced. Each sequence contains two constant regions at their extremities to allow PCR amplification, flanking a random region of *n* nucleotides (*n* ranging typically between 20 and 60). Usually, the library contains up to 10^14^–10^15^ different sequences. (2) These sequences are then submitted to an in vitro selection procedure in order to separate and predominantly amplify aptamers rather than the other sequences. The selection can be based on an affinity for a specific target or a catalytic activity.

Up to now, the library of the last round of SELEX was subjected to cloning and Sanger sequencing. Generally, a hundred of clones were sequenced revealing few sequences that were predominantly amplified. Therefore, a sufficiently large number of rounds were necessary to amplify aptamers up to a level that can be significantly measured by such low throughput sequencing. Recently, High-Throughput Sequencing (HTS) has been used to replace this approach. This technique, which already enables remarkable progress for transcriptomic and genomic analyses [6,7,8] and it is expected to dramatically change the way of performing molecular evolution processes.

**Figure 1:**
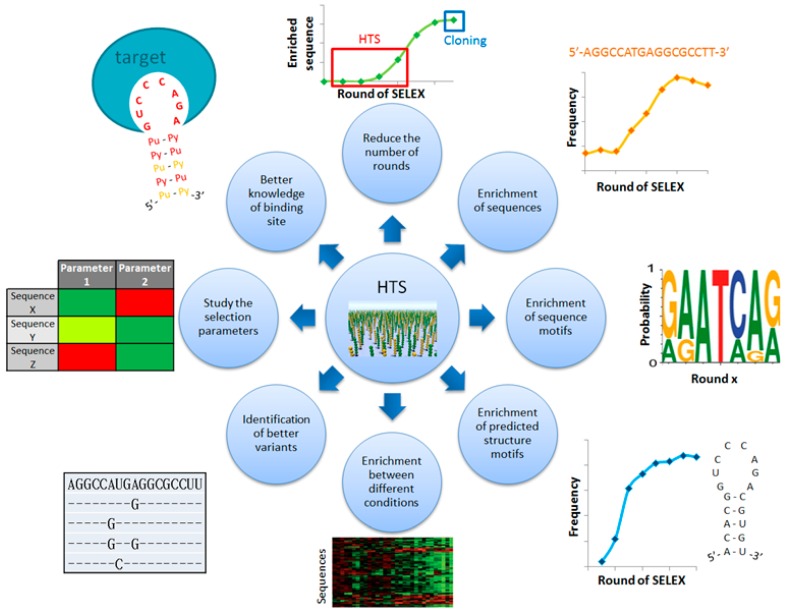
Improvements of aptamers identification and characterization with the use of High-Throughput Sequencing. HTS analysis can investigate faster the enrichment of (sub-)sequences or predicted (sub-)structures. Variants of a same family sequence can be compared in order to extract mutants with higher affinity than the most abundant sequence. It is possible to more precisely characterize the interaction between aptamers and their targets by analyzing different conditions of selection (for instance, incubation with target’s variants or varying the composition of binding buffer). It is also possible to find aptamers specific for a defined condition. Finally, the impact of each selection parameter can be studied which could help to improve the SELEX experiments in the future.

Indeed, HTS allows analyzing millions of sequences from all the rounds of a SELEX and opens new ways to better identify aptamers (Figure 1). Thus, it is now possible to detect enriched sequences at very low percentage (below 1%). As a consequence, enriched sequences can be observed with fewer selection rounds. Moreover, the high number of analyzed sequences ensures a statistically robust identification of enriched primary/secondary sequence motifs. This robustness also permits comparing rounds of SELEX with different conditions of selection in order to determine aptamers with specific skills or to better characterize the binding site of a known aptamer. Additionally, the mutational landscape can be explored to find the better variants of an aptamer family. The effect of selection parameters can also be studied to optimize the SELEX process. However, HTS requires manipulating very high amount of data up to several gigabytes, which is not supported by classical analysis software with low input capacity. Furthermore, there are several ways to analyze this data studying the enrichment of primary sequences and/or predicted secondary structure motifs. Therefore, the development of new bioinformatics approaches has been increasing in the past few years. This review presents these methods and how they improve the identification and characterization of aptamers.

## 2. Preparation of Libraries for HTS

Different technologies could be used to perform HTS and are provided by different suppliers including Illumina, ThermoFisher Scientific and Roche. These technologies have already been reviewed [7,9,10]. In most cases, “adapter” sequences have to be linked at the extremity of sequences by ligation or PCR amplification [11,12,13]. These adapters will be used to amplify the sequences and attach them to a sequencing support before sequencing (Flowcell, beads...). Several libraries could be mixed and sequenced in parallel, but in that case a supplementary “indexing” sequence has to be linked to each library in order to discriminate them.

## 3. Identification of Primary Sequence Motifs

One of the key advantages of HTS has been demonstrated in 2010 by Lorenz et al. They performed SELEX experiments to identify genomic aptamers that can bind to a RNA binding protein of *E. coli* named Hfq [14]. Ten rounds of this genomic SELEX were performed with a RNA library composed with *E.coli* genome fragments from 50 to 500 nucleotides. Sanger sequencing was used to analyze 170 clones derived from rounds 8 and 9. To investigate whether in vitro-selected sequences bind Hfq, some of these sequences were evaluated by yeast three hybrid system. All investigated clones were able to bind to Hfq. However, none was already known as Hfq-binding RNAs. The library of round 9 was further studied by HTS in order to analyze sequences with lower frequency. 8865 sequences were obtained and mapped to the *E. coli* genome. Then, sequences that overlapped were grouped into clusters. 1522 individual clusters were obtained and 94 of them were known as Hfq-regulated genes from previous micro array analysis. Unexpectedly, it was demonstrated that these clusters are four-fold more frequent on the antisense strand of protein coding genes than on the sense strand. Furthermore, a consensus motif could be predicted using MEME software and this motif was validated with DMS footprint experiments.

Another demonstration of the HTS sequencing usefulness was also presented in 2010 using SELEX to identify the DNA binding motifs that are recognized by transcription factors (TFs). Transcription factors (TFs) control when and where proteins are expressed inside cells. Therefore, it is important to know their DNA-binding specificity for a comprehensive understanding of gene regulation as well as for promoter engineering in biotechnological applications. Since its origin, several SELEX experiments have been conducted to identify the DNA binding motifs that are recognized by a transcription factor (TF) [15]. However, SELEX with classical low throughput sequencing can only provide rough initial models that need to be subsequently refined by others experiments such as competitive electrophoretic mobility shift assay or microwell based binding assays. Jolma et al. demonstrated that such SELEX experiments could be dramatically improved using HTS [16]. In their study, five rounds of SELEX were performed against 19 different transcription factors (TFs). The libraries of every SELEX were analyzed at each round using high-throughput multiplexed sequencing. Compared to classical sequencing, the identified DNA motifs were based on 100 to 1000-fold higher number of sequences. An average of 37,590 sequences was analyzed from each round of SELEX using a computational pipeline called ‘IniMotif’. The frequency of all subsequences of 5–11 nucleotides from the 14-mer dsDNA library was counted. Then, the most enriched subsequences at each length were compared to all others with a hamming distance (number of substitution between two subsequences) to identify clusters and obtain a consensus motif of each round. The validation of the method was achieved by comparison with already identified motifs. Interestingly, the large amount of data allows the analysis of composite/dimeric sites and reveals unexpected dimeric modes of binding for several TFs. The same group further used this method to analyze the sequence-specific binding of hundreds TFs [17].

A similar approach to identify binding sites of TFs has been developed by Reiss et al. using genomic SELEX [18]. For that purpose, another software was developed and called Transcription Factor Analysis using SELEX with High-Throughput sequencing (“TFAST”) [19]. TFAST counts and aligns millions of sequences to a genome in order to identify peaks that correspond to genomic sequences predominantly amplified during the SELEX. Evolution of peaks is analyzed at each round of selection and at the end a score is calculated to discriminate peaks with a higher probability to contain a binding motif from dummy sequences. The score is based on the rate of enrichment, with different weight applied in each round to take into account the progression in the SELEX. This method was applied to analyze a genomic SELEX of four rounds against a TF of *E. coli* named PapX. Around 24 million sequences were analyzed per round. This huge amount of data covered 400 times the CFT073 genome of *E. coli*. A total of 457 peaks provided a better enrichment throughout the different cycles and were used to identify a consensus motif using MEME software. This study revealed that PapX interacts with the flhDC promoter to repress the expression of flagellar genes.

The strategies described previously are useful to identify natural interactions of nucleic acids with proteins that are known to bind DNA or RNA. However, these methods are difficult to apply for other SELEX that are realized to identify artificial binders commonly named aptamers. In such cases, there is no a priori knowledge of the lengths and the structures of nucleic acids that could be selected during SELEX. Therefore, the analysis of sequencing should be more global and it should take into account that several sequences with different foldings could be selected to bind the same target. The first step to treat HTS sequencing of such SELEX is to recover the sequences between primer regions and to count their frequency at several rounds of SELEX. While several programs dedicated to analyze SELEX sequencing integrate these steps, it can be done using the well-known platform called Galaxy Project [20,21]. Galaxy Project provides basic bioinformatics tools for everyone without a background in informatics. This platform can handle HTS data in an easy way using different functions as building blocks and each function can be sequentially added into a pipeline. For the analysis of SELEX experiment, Thiel et al. proposed a pipeline to (1) remove the constant regions of each sequence; (2) apply a quality filter for the sequencing; and (3) count the copy number of each sequence in a round of SELEX [22]. Moreover, they explained how to compile these results to have a table presenting the evolution of the copy number of each sequence at every round of SELEX. Although such a pipeline could be useful for preliminary analysis, other tools mandatory for a deeper analysis are not available in Galaxy Project mainly because it is a platform addressed to genomic projects.

To solve this drawback, several other programs have been developed to perform the first steps previously described and to cluster sequences based on their similarities. Using low throughput data, the best global alignment could be searched using different programs such as clustalX/Omega. However, they cannot manage the high amount of data generated by HTS. Thus, several other approaches have been developed to cluster sequences using the most abundant sequences as ‘seeds’ and assuming that these sequences have a higher chance to contain aptamers. “FASTAptamer” [23], “AptaCluster” [24] and “PATTERNITY-seq” (personal communication [25]) are based on this strategy although they adopt different methods of clustering. FASTAptamer and PATTERNITY-seq use the Levenshtein distance to group sequences around sequence seeds. Levenshtein distance takes into account all kinds of mutation (substitution/insertion/deletion) to compare sequences even at different size. The use of this distance to form clusters makes sense in SELEX experiment where it is possible to observe enrichment of sequences with more or less nucleotides than expected. However, a filter based on the size of the random part has to be used in order to remove sequence too long or too short. Moreover, the computation time could be very long if there are millions of sequences to compare because this method uses matrix distance. To solve this drawback, a threshold is often used to analyze only sequences that are higher than a defined frequency. This parameter decreases the computation time and avoids analyzing sequences that are not significantly enriched during SELEX. Additionally, an option exists with FASTAptamer to search for a known primary motif in the pool.

Another way of clustering has been used in the software AptaCluster based on a Local Sensitive Hashing (LSH) method that allow comparing sequences with a reduced number of dimensions. The hashing function randomly chooses a defined number of dimensions (for instance, it could be a string of nucleotides) to compare and group sequences that have a similar LSH values based on hamming distance. Then, a k-mer distance is applied in each group, on the entire length of sequences, to define more precisely clusters. k-mers refer to all the possible subsequences (of length k) from a sequence. Such a method is fast since it does not require building a matrix distance in contrast to Levenshtein distance. However, several pitfalls exist: (1) hamming distance does not take into account insertion and deletion. (2) The method cannot be used with sequences of different size. Therefore, it is necessary to filter data based on the length of sequences before using these methods. (3) Manual parameters of LSH method such as number of used dimensions and number of hashing function iterations have to be correctly adjusted for each different SELEX to reduce the probability of wrong pre-grouping as much as possible.

Finally, another approach of clustering has been implemented by the company AptaIT GmbH using the software “COMPAS” (COMmon PAtternS) [26]. COMPAS can cluster sequences based on k-mer distance without LSH or based on the criteria of Shannon’s entropy. However, the company does not provide details on their method.

## 4. Identification of Secondary Structure Motifs

Clustering based on the primary sequence is important for the analysis of SELEX, but it is well known that aptamers recognize their target by adopting a precise 3D conformation (e.g., kissing complex, A-minor motif...). Therefore, it is important to study the enrichment of sequences in the context of a structure. While 3D structures of nucleic acids are still difficult to predict, several software can be used to predict their secondary structures (e.g., stem loop, bulge, internal loop) such as RNAalifold [27] or Mfold [28]. However, such programs are not designed to manage high throughput data. To solve this drawback, Thiel et al use a series of software to analyze height rounds of a SELEX that were performed to identify aptamers able to internalize inside vascular smooth muscle (VSM) cells [29]. After removing all sequences that did not have more than three copies and present at least in two rounds, the frequency of 2312 unique sequences was measured at each round. These sequences were clustered in families using two methods. The first approach used a Levenshtein distance as previously described. For the second method, a program (named “process.seqs”) was developed using RNAfold and RNAdistance from the Vienna RNA Package [30]. It first predicts the most likely structure of all sequences and then determines the edit/tree distance of all sequences/structures. Based on these two clustering methods, 32 sequences were selected for internalization studies. Several aptamers showed a better internalization in VSM cells compared to a control sequence. Noticeably, the best-internalized sequence was only identified by secondary structure clustering. 

Another bioinformatics pipeline was used by Ditzler et al. to find a better RNA inhibitor of HIV reverse transcriptase reanalyzing a SELEX experiment previously performed in 1996 [31,32]. Around 1,000,000 sequences from round 14 were analyzed. First, the copy number of each unique sequence was counted before clustering them using Levenshtein distance from seed sequences. Then, the 5000 most abundant clusters were extracted and sequentially used by different programs: (1) Mafft [33] aligned sequences in each cluster; (2) this alignment is used by RNAalifold from the Vienna RNA package [30] to build a consensus predicted secondary structure for each cluster, based on conservation and co-variation; (3) this information is then further used by the software Infernal [34] to construct covariance models (CMs) among the 5000 clusters. This pipeline allows to group different sequences that share enriched sub-sequence-structures. Five distinct converged structural motifs were identified. Among them, three classes of pseudoknots represent 93.8% of the pool. These structures have been already identified from the previous analysis using classical cloning and sequencing methods. However, HTS analysis provided two new stem loop structure motifs including an asymmetric internal loop structure ((6/5)AL) that represents only 2.9% of the pool. This new structure was impossible to detect with low throughput sequencing but, interestingly, it demonstrated a strongest inhibition of the reverse transcriptase.

“AptaTrace” is another software that has been recently developed to identify structure motifs that are enriched during the SELEX [35]. Using SFOLD [36], predicted secondary structures are built for all sequences and a secondary structure probability profile is made for each nucleotide in the sequences. This information is used to provide a predicted structure context to each k-mer of a defined length. For instance, it allows predicting if a specific k-mer has the highest chance to be found in a loop, a bulge, a base pair, etc. Then, the evolution of the predicted structure for each k-mer in each round is performed and k-mers are ranked based on significant enrichment in a specific predicted structure. Furthermore, a clustering between k-mers enriched in the same predicted structure context is performed to identify consensus motifs. To validate AptaTrace, Dao et al. used data from a cell-SELEX previously performed to identify aptamers against the C-C chemokine receptor type 7 (CCR7) [37]. A CUGUG motif highly represented in an apical loop structure was found and two mutants of this family (C1-A and C2-A) demonstrated binding by flow cytometry assays. However, these two sequences were already identified by a classical approach since they were the most abundant sequences in the cell-SELEX. Therefore, it is difficult to know whether such a method can be used if aptamer sequences are not fully enriched.

Another software, called “APTANI”, proposes to use secondary structure information [38]. First, secondary structures are predicted using RNAsubopt from the Vienna RNA package [30]. Then all suboptimal secondary structures within a user defined energy range above a minimum free energy (MFE) threshold are recovered. Based on these predicted structures, four kinds of sub-structures are considered (apical loops, bulge loops (either right or left) and intra-strand loops). Every sub-structure motif is aligned using Clustal Omega [39] in order to obtain a consensus sequence. Therefore, a score is calculated for every sequence based on its similarity to the consensus of every sub-structures. The more the sequence matches several sub-structures, the higher the score. This method was validated using a SELEX against IL4Ra [40]. The previously discovered aptamer (C1.42) was identified by APTANI after only one round while the method used in the first paper identified it after five rounds.

It is important to note that most programs that implement structure prediction to analyze HTS are currently focusing on stem loop prediction. However, other structure motifs are often found in aptamers such as G-quadruplex. Therefore, these programs may be completed using other prediction models in order to add this important class of structure. For instance, Stegle et al. develop a model to predict G-quadruplex stability [41].

## 5. Study of Evolution

Until now, it was impossible to use low throughput sequencing to understand how binders are enriched during SELEX. Such analysis is now possible using HTS since it can easily analyze millions of sequences in parallel from every round. Therefore, it can provide important information on what was seen as a black box for many years. In this way, Schutze et al. studied the evolution of a DNA library during 10 rounds of SELEX against streptavidin [42]. To monitor the evolution of the library, the number of sequences at one copy was counted at every round. This number started to dramatically decrease after three rounds, demonstrating that some sequences started to be enriched. Moreover, most of sequences at one copy after five rounds were identified as mutants of the strongly enriched sequences, suggesting they were generated from mutation introduced by polymerases. Interestingly, the authors observed that the most abundant sequences in the final round did not directly correlate with the strongest binding behavior. Furthermore, the frequency of one of the best binders (R10#17) was found to increase in the pool until round 7 before decreasing. This decrease is a consequence of further amplification of other sequences with weak binding.

Berezhnoy et al. confirmed this observation with a SELEX against murine IL-10 receptor [43]. The best binders were identified by high throughput sequencing after five rounds, progressively disappeared while weak binders were more frequents at the end of the SELEX (16 rounds). Thus, it seems that sequences could be amplified during SELEX based on other criteria than binding for the expected target. Such unwanted selection was also observed in a cell-SELEX against CHO-K1 cells overexpressing endothelin type B receptor [11]. The wild type CHO-K1 cell line was used at every round in negative selection steps to discard any aptamer that could be selected against other membrane proteins than ETBR. Analyzing the frequency of sequences at every round demonstrated that several sequences were progressively amplified during this SELEX. However, only few of them were able to bind cells and no one was specific against ETBR. Therefore, it demonstrated that sequences could be enriched during SELEX without any binding behavior or with specificity to other targets than expected despite the negative selection steps.

The observation of enriched sequences without any binding behavior is often described in the literature. Most of the time, it is suggested that sequences that have a higher capacity to be amplified by polymerases could be amplified during SELEX even if they do not bind the target. Zimmermann et al. studied this potential bias inside a genomic SELEX [44]. They used the same starting library as Lorenz et al. who performed a SELEX against the RNA binding protein Hfq (previously described). Ten rounds of SELEX without incubation with the target were performed. As a consequence, they amplified at every round the sequences that are adapted to be amplified by the method in the absence of the target. As for the SELEX against Hfq, this SELEX demonstrated a clear evolution of the pool toward sequences with less stable predicted structures and shorter lengths. This observation agrees with the idea that highly structured templates are more difficult to amplify than a weak structure. However, no enrichment of particular sequences was observed during this ‘neutral’ SELEX in contrast to the one with Hfq target.

The effect of PCR on the SELEX process has also been study using HTS by Takahashi et al. [37]. Two cell-SELEX were performed against CCR7. The first SELEX used classical PCR and leaded rapidly to the amplification of a sequence (C-1A) that represented 85.97% of the pool after five rounds. In contrast, droplet digital PCR (ddPCR) was used in the second SELEX and seemed to provide a slower evolution of the pool. Indeed, the same sequence (C-1A) was the most enriched sequence after five rounds but it represented only 18.81% of the pool.

In order to discriminate the enrichment that originates from the binding to a target from other factors (binding to matrix support, PCR bias...), Jiang et al. developed another software called “MPBind” [45]. MPBind calculates a meta-Z score for each sequence taking into account several parameters. Basically, the highest score will be attributed to sequences that contain k-mers that are much more enriched in the pool selected with a target than in the pool selected in the same condition without the target. The method was evaluated using a cell-SELEX of five rounds against human embryonic stem (hES) cells. Nineteen sequences were selected with a large range in their Meta-Z-score. Almost all of the candidates with a positive meta-Z-score showed an affinity to the targeted cells. In contrast, all the candidates with a negative meta-Z-score did not show any binding. However, when MPBind was used by another lab to analyze another SELEX against the ovarian cancer marker HE4, the correlation was weaker between the score and the affinity of the sequences [46]. Indeed, the best binders showed a negative Z-score.

## 6. Speed up of SELEX Process

One of the most expectations regarding HTS is to reduce the number of in vitro selection rounds and at last, to speed up the SELEX process. This is particularly true for libraries with short random regions. For instance, Dausse et al. performed a SELEX against the XBP1 RNA using a mixture of two pre-structured stem loop RNA libraries containing either 10 or 11 random nucleotides in the loop [47]. Therefore, the starting pools contain around 5 × 10^6^ sequences. Six rounds of selection were performed on a Surface Plasmon Resonance instrument to evaluate the affinity of the library. A shift in the SPR signal was observed after four rounds, suggesting that the pool was enriched in binders. The library of round 6 was sequenced by Sanger sequencing method. A consensus of seven nucleotides was shared by several sequences and was quite complementary to the 5′ loop of XBP1. Almost all of them showed a fast association with the target and the best sequence showed a Kd equals to 8 nM. The library was also sequenced by HTS after two and four rounds. Among the millions of sequences analyzed, the motif was found at 4.5% and 43% at rounds 2 and 4, respectively. Therefore, even if no SPR binding signal was detected after two rounds of selection, HTS was able to identify enriched motifs.

Other studies performed only one round of in vitro selection. For instance, Kupakuwana et al. realized a single round of counter-selection/selection against human α-thrombin starting from a pre-structured stem loop DNA library containing 15 random nucleotides in the loop [48]. 56,000 copies of each random sequence were supposed to be present in the 100 pmol of the starting library. After the selection, 1,728,220 unique sequences were identified. 92.5% of them presented one copy but 107 sequences were present more than 10 times in the post-selection pool. These 107 sequences were grouped in two families based on alignment with ClustalX. The first family contained an already known G-quadruplex aptamer (Thb1). This aptamer was the most amplified sequence (46,444 copies) in the pool. The second family possessed the second most amplified sequence (Carb1, 29,405 copies) but it showed an affinity for hexose sugars that are present in the partitioning matrix. Interestingly, two other in vitro selections, performed with increasing concentration of hexose in the selection, led to an enrichment of the Carb1 family instead of Thb1 family. This result highlighted how HTS could be used to study the impact of the presence of different targets during the selection. All together, these studies demonstrate that HTS could be used to discriminate sequences that are amplified as a consequence of binding to a target of interest from those that are amplified due to other capabilities.

## 7. Study of Specificity and Fitness Landscapes

Another advantage of HTS is to improve the affinity or the specificity of aptamers. Ditzler et al. used such strategy to select aptamers with a slower dissociation rate [32]. Following the study previously described to identify RNA aptamers against the reverse transcriptase (RT) of HIV, they compared the frequency of sequences in a pool that underwent one round of SELEX with increasing stringency. After 14 rounds of a classical SELEX, the library was split and used in parallel for one round of SELEX in different conditions. First, the selection was performed with or without RT in order to discriminate the sequences that could be enriched due to a higher level of protein-independent background binding to the filter and not a higher affinity for RT. Then, the selection was performed in the presence or absence of an excess of a DNA aptamer competitor that cannot be amplified but could compete for the binding of RNA aptamers. Several conditions were compared in parallel with increasing time in the presence of competitor (0, 15, 60, and 240 s). Based on classical SELEX, an aptamer motif with an asymmetric loop ((6/5) AL) was previously identified as a better inhibitor of RT although it was not predominantly amplified compared to aptamers with pseudoknot structures. Interestingly, the more the stringency was increased, the more the frequency of the (6/5) AL motif increased in the pool. Ditzler et al. also demonstrated that HTS can provide the sequencing depth necessary to study in more details the variants of (6/5) AL motif. It is well known that mutations can occur by polymerases during SELEX, leading to the emergence of variants that may have better affinity than the originate aptamers. The comparison of the evolution of these variants can provide important information on the binding site of aptamers. Therefore, a mutant of the (6/5) AL motif was highlighted due to its higher enrichment during the increase of stringency compared to its enrichment in absence of target. This new mutant was at a very low frequency in the pool but it strongly inhibited the RT, even more than the dominant mutant of (6/5) AL motif.

The study of mutation incidence using HTS was even more explored by Pitt et al. who analyzed a doped-SELEX with a dedicated software “SEWAL” (Sequence Evolution With Adaptive Landscape) [49]. A doped-SELEX uses as a starting pool a well-characterized in vitro selected nucleic acid structure that is mutagenized and subjected to a reselection. SEWAL builds an empirical fitness landscape that links genotypes and the enrichment during a selection process. Such analysis is only possible with HTS, because it needs to access a very large experimental sequence space. Sequences are plotted in a 3D graph where their distance to a sequence seed (the parent of the doped library) is in the *x*-axis, their distance to a control sequence is in the *z*-axis, and their enrichment during the selection is in *y*-axis. Fourth information could be added on the 3D scatter plot by changing the color of spots. For example, it is possible to change the color to highlight sequences that contain a particular nucleotide at one position. This software was used to study an RNA class II ligase ribozyme [50]. A doped pool from this ribozyme was subjected to one round of in vitro selection with increasing stringency. The 3D graph showed an artificial fitness landscape that was relatively steep and narrow. It demonstrates that most of the individual mutations greatly decreased the functionality of the ribozyme. Furthermore, it identified some positions that appeared to be selectively neutral.

Another tool, named “AptaMut”, was developed by Hoinka et al. to study the evolution of mutants during a SELEX using HTS [51]. This software first needs to cluster sequences. Then, for each family, the enrichment between two rounds of the most abundant sequence is compared to the other ones. If a variant has the same enrichment of the most abundant sequence, it should contain mutations that have no impact on the affinity. In contrast, if a variant has a higher or lower enrichment, it should contain mutations that provide a higher or lower affinity, respectively. A score is calculated based on this comparison to highlight the variants that could provide a better affinity than the most abundant sequences. Most of the time, the most abundant sequences are chosen for binding evaluation while variants are ignored. AptaMut was evaluated with a SELEX against Interleukin 10 receptor alpha chain. Several variants from three families were tested for binding and most of them demonstrated a similar affinity than the most abundant sequence of their family. Nevertheless, two of these variants provided a two times better affinity. Therefore, although the score was not perfectly correlated to the affinity, it can identify better aptamers than the most abundant sequence.

Additionally, to improve the identification of aptamers with higher affinities, HTS can be used to select aptamers with a desired specificity or to study their binding sites. For instance, Dupont et al. demonstrated that HTS can be used to provide important knowledge about aptamer binding sites and to identify aptamers against different sites of a target protein [52]. For that purpose, they used a 2′-fluoropyrimidine RNA library originated for five rounds of SELEX against the serpin plasminogen activator inhibitor 1 (PAI-1). This library was split and used in parallel for a single round of in vitro selection to different PAI-1 variants (wild type and single-residue alanine mutants) and conformers (active and latent form) as well as PAI-1 presented by different antibodies or the natural extracellular matrix protein, Vitronectin (VN). Pools from each condition were further analyzed by HTS. In a previous study, two aptamers were already identified (paionap-5 and paionap-40) to interact with the Vitronectin binding site of PAI-1. The first one interacts with Arg78, Lys82, Phe116, and Arg120 of PAI-1 while the second one recognizes also Lys124 [53]. These previous identified aptamers were represented at 0.405% and 0.069% for paionap-5 and -40 in round 5, respectively. After one round of selection against the wild type target, enrichment factor (EF wt) of both sequences increased. However, when it is a target’s variant, the enrichment factor (EF variant) changed, depending on the impact of this mutation in the binding of the aptamer. Therefore, they confirmed that both aptamers do not interact with the position 71 of PAI-1 while mutation on positions 116 and 120 strongly decrease their binding. EF ratio was also used to predict successfully that paionap-5 is able to bind active PAI-1 as well as latent PAI-1, which is not the case for paionap-40. On the other hand, as expected, it was predicted that both aptamers could not bind to the target when Vitronectin is already bound to PAI-1. Nevertheless, by analyzing EF with the round against PAI-1/Vitronectin complex, they found new aptamers that can bind to PAI-1 without competition with Vitronectin. Two of these aptamers were present at 22 and two copies in the pre-enriched pool and became the third and the 230th most frequent sequences of the branched selection against PAI-1:Vitronectin. In conclusion, this study clearly demonstrated that HTS analysis of multiple in vitro selection conditions could provide important information on structural features that are involved in the affinities and the specificities of RNA-protein interactions.

Such a strategy was also used by Meyer et al. to discriminate a DNA aptamer specific of the murine c-kit cell surface receptor from sequences that were enriched without affinity for the target [54]. During their cell-SELEX, they used for the selection a lymphoblastoma cell line (BJAB) transformed to over-express c-kit and the wild-type cell line for counter-selection. After five rounds, the library was split to perform a sixth selection round on both cell lines. Analyzing around 18.5 million sequences for both pools, they identified several sequences with a higher abundance from selection on c-kit expressing cells compared to wild-type cells. They tested the binding of 10 aptamers with a higher ratio between the two cell lines and identified four aptamers with a higher binding on c-kit expressing cells. A similar strategy was also used by Levay et al. where a parallel selection was performed after three rounds in order to identify aptamers against hIL-10RA [55]. Noticeably, they also performed and obtained results in another parallel selection to find aptamers that can bind to both the human and the murine form of IL-10RA.

## 8. Conclusions

High-throughput sequencing is expected to replace cloning and Sanger sequencing. However, the use of such technology is not straightforward and it can be costly and time consuming [56,57]. Therefore, it is difficult to know how it will be routinely used in the future SELEX experiments. For instance, many studies described in the present review are expensive since they require analyzing very large numbers of sequences from multiple rounds of SELEX. Nevertheless, HTS can certainly be used to reduce the number of selection rounds of a SELEX or to reduce the number of post-SELEX experiments needed to improve aptamers. For example, the high resolution of HTS provides access to the mutational landscape of an aptamer. This information could be used to find better binders or to find the ones that can bind in a specific condition. Thus, it will be mandatory to define a good balance between the cost and the benefit of using HTS.

Currently, several programs with different strategies have been developed to analyze HTS data from SELEX (summarized in Table 1). Some can be freely downloaded and used, but most of the time they require expertise in bioinformatics. Notably, it is mandatory to understand the different parameters that are used to limit false positive results. In contrast, some other tools are available through paid services that guarantee technical support provided by companies or academic platforms. 

One drawback in the field is a lack of comparison between the different programs on the same batch of data. Moreover, most of HTS data from SELEX experiments are not currently deposited in public databases such as Sequence Read Archive (SRA) and, as a consequence, they are not available to the research community. Nevertheless, it is expected that the efficiency of all these different approaches will be evaluated in the future in order to identify the best ones. 

Finally, several teams are already working to develop high throughput binding evaluation of sequences. Such approaches could be useful to evaluate or complement HTS analyses. For instance, a method called “HAPIscreen” is able to evaluate the binding of aptamers in 384-well microplate format [58], another method has also been developed to screen around 15,000 sequences thanks to micro-array technology [59]. However, the most exciting solution could be to perform high throughput binding and sequencing on the same instrument. Such an approach has recently been proposed in an Illumina sequencing apparatus where sequences were first sequenced before being evaluated for binding to a fluorescently labeled protein [60,61].

## Figures and Tables

**Table 1:** Comparison of several programs dedicated to HTS analysis for SELEX experiments.

Name	System	Rounds Analyzed	Clustering Based on Primary Sequence	Clustering Based on Secondary Predicted Structure	References
**IniMotif**	Not determined	Several	Clustering of the most enriched subsequences using hamming distance.	-	[16]
**TFAST**	Mac/Linux/PC	Several	Alignment on a genome	-	[19]
**FASTAptamer**	Mac/Linux; Galaxy web platform	2	Levenshtein distance on sequence ‘seeds’; possibility to look for a known motif	-	[23]
**AptaCluster**	Linux	Several	LSH method followed by k-mer distance on sequence ‘seeds’	-	[24]
**PATTERNITY-seq**	Available through services	Several	Levenshtein distance on sequence ‘seeds’	Look for predicted structure motifs shared by several primary clusters	[25]
**COMPAS**	Available through services	Several	k-mer distance or shannon’s information entropy	Can detect stem loops shared by several primary clusters	[26]
**AptaTrace**	Mac/Linux/PC	Several	Look for enrichment of k-mer with a predicted structure; then, primary alignment of k-mers with the same predicted structure is realized to form clusters		[35]
**APTANI**	Linux	Several	Look for four kinds of sub-structures in each sequence; primary alignment of the sub-structures to form clusters		[38]
**MPBind**	Mac/Linux	2	Rank sequences based on k-mer enrichment	-	[45]
**SEWAL**	Mac	Several	Hamming distance on two sequences (seed and control) to obtain (x,y) coordinates of all sequences to build the empirical ‘landscape’	-	[49]
**AptaMut**	Linux	2	Rank variants of a primary sequence family based on their enrichment	-	[51]
